# Real-World Meditation App Engagement: Longitudinal Study of the Medito Meditation App

**DOI:** 10.2196/79366

**Published:** 2026-06-08

**Authors:** Julia Adams, Jonathan Davies, Prai Wattanatakulchat, Julieta Galante, Simon D'Alfonso, Nicholas T Van Dam

**Affiliations:** 1 Contemplative Studies Centre, Melbourne School of Psychological Sciences, The University of Melbourne, 700 Swanson St, Melbourne, 3066, Australia, + 64 2904309289; 2 Centre for Innovation in Pain and Health Research, The University of Queensland, Brisbane, Australia; 3 School of Information and Computing Systems, Melbourne School of Psychological Sciences, The University of Melbourne, Melbourne, Australia

**Keywords:** meditation, mindfulness, engagement, app, meditation apps, digital mental health interventions, behavior change

## Abstract

**Background:**

Meditation apps are increasingly popular but face significant engagement challenges. Most research does not meaningfully capture real-world engagement or associated user characteristics. Engagement patterns and reasons for engaging or disengaging remain relatively unexplored.

**Objective:**

This study aimed to examine Medito app user engagement over the first 30 days after download and how intended use, demographics, user traits, and mental health factors predict engagement.

**Methods:**

A prospective online survey was conducted among 668 Medito app users from 30 countries. Factors assessed included demographic factors (eg, age, sex, education, employment, and country of residence); user factors (eg, number of apps tried, hours of experience, meditation-related adverse events, expectations, readiness to change, and personality); and mental health factors (eg, quality of life, perceived stress, psychological distress, well-being, and satisfaction with life). Detailed engagement data included days of use, meditations completed, app opens, and minutes of use obtained via a data-sharing agreement with Medito. Minutes of use in the first 30 days after download served as the main outcome variable.

**Results:**

App use was relatively low, with 50% (328/655) of users engaging for a total of 16 minutes or less in the first month after download (median 16.11, IQR 0‐74.51 min). Fewer than 20% (124/655, 18.86%) of users continued using the app after 14 days. Intended use (mean 418.56, SD 472.5) significantly exceeded actual use (mean 70.02*,* SD 176.81; *d*=0.710; *P*<.001). In terms of user factors, expectation match (ie, extent to which outcomes from the app matched initial expectations; ρ*=*0.214; *P*=.002), expectations for anxiety (ρ=0.102; *P*=.01), expectations for attention or focus (ρ=0.091; *P*=.02), and conscientiousness (ρ=0.124; *P*=.003) were associated with higher engagement. Neuroticism was negatively associated with engagement (ρ=−0.103; *P*=.010). For mental health factors, satisfaction with life (ρ=0.123; *P*=.002) and well-being (ρ=0.135, *P*<.001) were associated with higher engagement, while perceived stress (ρ=−0.107; *P*=.007), psychological distress (ρ=−0.138, *P*<.001)*,* and quality of life (ρ=−0.100; *P*=.011) were associated with lower engagement. Only readiness to change showed unique associations with higher engagement (semipartial *r*=0.156; *P*<.001). Regression analysis showed that only perceived stress predicted higher engagement (β=.020; *P*=.04). However, when mental health was included as a single component, expectations for anxiety (β=.015; *P*=.049) and readiness to change (β=.011; *P*=.048) predicted greater engagement, and mental ill health predicted lower engagement (β=−0.008; *P*=.049).

**Conclusions:**

Overall, app engagement is generally quite low. Acute stress motivated meditation app use, while chronic stress disrupted it. Engagement is optimal when experiences match expectations and users are prepared to make a change. More transparency is necessary in the promotion of meditation apps so that users have a realistic understanding of the time and effort required to achieve benefits.

## Introduction

### Background

Mental health is a global issue, with 1 billion people living with a mental health disorder—a 50% increase since 1990 [1]. Mental health interventions require 10 to 15 times more resources (eg, medicines, personnel, and equipment) than other health interventions to impact disability-adjusted life years (a measure of the burden of disease through years lost to death and disability), thereby straining limited resources [2]. Smartphone-based meditation apps offer a scalable solution to provide general mental health support [3]. However, with fewer than 10% of individuals who download an app regularly using it beyond 1 week [4] and dose-response examinations suggesting high minimum practice requirements [5,6], most users are unlikely to achieve clinically significant improvements as a result of using a meditation app. A better understanding of what predicts engagement with meditation apps would help support overall use and the targeting of users most likely to benefit. While engagement rates in meditation apps and associated factors have been explored [4,7], to date, limited research has examined how baseline characteristics predict meditation app use over time, which could inform our understanding of which individuals may benefit most from these apps.

### Mindfulness-Based Programs, Apps, and Efficacy

Meditation apps make up the majority of mental health apps [8], with the 2 leading apps, Headspace (Headspace Inc) and Calm (Calm Inc), collectively accounting for 70% of the market share in the mental health app sector [9]. Several evidence-based not-for-profit apps have emerged in recent years, such as the Medito app (Medito Foundation) [10]. Unlike traditional mindfulness-based programs (MBPs), which involve group and individual sessions that average 6 hours per week [11,12], most meditation apps involve no human support and have a lower suggested time commitment [5]. While MBPs show moderate improvements in a variety of mental health outcomes [8,13], apps produce significant but smaller effects compared with passive controls [14,15]. App efficacy is undermined by higher attrition rates in randomized controlled trials of apps than those of face-to-face MBPs (see section “*Attrition, Adherence, and Engagement*”), with most users abandoning apps within a week of download [4].

### Attrition, Adherence, and Engagement

A challenge in digital meditation research is that engagement is not reported in one-quarter of studies [16] and, where noted, is largely attrition rather than meaningful app use. Engagement measures the amount, frequency, and duration of use, while attrition refers to abandonment or noncompletion rates in an intervention or study. Traditional in-person MBPs have low attrition from study measures (13%‐20%) [17], while attrition from study measures in meditation app randomized controlled trials is approximately 42%, with 90% attrition from application use after a week in naturalistic populations [4,14]. Adherence refers to compliance with an intervention that has a clinically effective “dose” [18]. Adherence captures engagement beyond attrition but is less relevant in nonclinical meditation apps that have no empirically ascertained minimal dose [6]. In our previous analyses of retrospective use patterns, meditation apps were only used, on average, 2.5 times per month.

A further challenge is that digital meditation studies often rely on self-reported adherence or attrition, offering limited insight into use patterns, reasons for disengaging, or real-world use with lower motivation and minimal guidance. Intended and self-reported engagement are typically higher than actual engagement levels and reflect distinct constructs, with some findings suggesting no relationship between reported and actual smartphone use [4,19-21]. Previous work has explored real-world use patterns [4,19,20], predictors of use [7,14], and associations with engagement as well as the relationship between well-being and app use [22,23]. Despite this work exploring factors and use, little is known about which user characteristics predict sustained engagement during the critical first month of use. Understanding prospective indicators of early use patterns may reveal user trajectories, distinguishing the features of those who engage from those who disengage.

Our previous study examined factors associated with meditation app use in general [7], but the retrospective nature of the study limited our ability to identify causality and may have been affected by inaccurate recall [24-26]. Given the importance of consistent engagement in achieving meaningful outcomes [27] and gaps in engagement reporting, examining predictors of engagement with accurate, objective data during crucial habit-building stages [28] could provide valuable insights into what drives meaningful engagement in meditation apps. Prior work has captured retention rates for the first 30 days of use based on publicly available data. A 30-day period provides insight across typical large early drop-offs in app use. This time frame supports an understanding of both individuals who engage and those who disengage.

### Reasons for Engaging or Disengaging

Factors predicting app use often focus extensively on app characteristics alone [29] but have recently been extended to include sociodemographics, behavior change, and mental health factors. User factors that affect engagement are explorative but broadly reflect theories of habit formation and planned behavior [30,31].

#### User Factors: Sociodemographics

Sociodemographic factors may influence engagement in meditation apps. Older age and higher levels of education have been associated with greater engagement in meditation or mobile health apps [23,32], which may be due to poorer health outcomes or health literacy, although use to address health-related issues appears to be common [33,34]. Lower engagement has been found among minority groups in the United States [35,36]. Comparisons of engagement across countries are limited, although one cross-sectional survey found that residing in the United Kingdom was positively associated with engagement [7]. Most meditation app users have relatively high levels of income, although lower-income users may be more engaged in US samples [37,38]. Being female was found to be associated with lower engagement [7]. Barriers include difficulty finding a suitable space for practice and challenging thoughts [39,40]. Spiritual motivations for meditation also correlate with engagement [5]. Personality factors also predict engagement or cause meditation-related challenges [41], and the latter may result in discontinuation of practice [41]. Agreeableness, conscientiousness, openness, and neuroticism have all been found to predict engagement in meditation [37,42-45], although neuroticism has been linked to more perceived barriers to meditation [46,47].

#### User Factors: Expectations and Experiences of Progress in Behavior Change

Behavior change factors may also be relevant to meditation app engagement, such as the alignment of expectations and experience. In a prior cross-sectional study, expectations of efficacy (eg, for sleep, stress, and other outcomes) correlated with engagement [7]. Ongoing engagement is promoted by experiences of progress [39,48]. Failure of an intervention to meet expectations predicts (dis-)engagement more than matched expectations [49,50]. Unmet or low expectations reduce perceived usefulness, while matched or exceeded expectations positively influence opinion and behavior [51]. App design can influence expectations, as features such as goal setting and feedback increase perceived efficacy [52]. Fewer perceived barriers and a more positive perception of meditation predict engagement in both meditation in general and meditation apps [32,37,53]. Other factors linked to engagement include higher perceived effectiveness and a higher growth mindset [37].

#### Mental Health Factors

The relationship between baseline mental health and meditation app engagement remains unclear. Our preceding cross-sectional study found no clear association of engagement with mental health factors [7]. Prior studies suggest that higher anxiety and motivations for mental health are associated with lifetime use, although the relationship with engagement remains unclear [37]. Experiences of mental ill health can lead to more barriers or difficulty engaging in behavior change [54]. As people often use meditation apps for mental health reasons, research should further investigate how baseline mental health factors may contribute to engagement.

### Prospective Study Designs

Prospective study designs allow researchers to assess causality in engagement directly and measure baseline factors in real time. Prospective work is also beneficial for determining factors that fluctuate over time. Apps may improve mental health outcomes [8], which could positively or negatively reinforce app use. Additionally, as continual app users are likely to provide higher app ratings over time, understanding baseline expectations will provide insight into whether expectations for efficacy are significant in predicting use.

### This Study

This prospective study sought to examine the pattern of engagement seen in new users of the free not-for-profit meditation app, Medito, over the first 30 days of download. The study addressed the following questions, with the following hypotheses stated in the preregistration. Note that question 3 and hypothesis 3 are question 4 and hypothesis 4, respectively, in the preregistration. Preregistration question 3 and hypothesis 3 were not addressed in this manuscript.

- Question 1. What is the *pattern* of engagement in the Medito meditation app in the first 30 days after download?
- Hypothesis 1. Engagement rates will decrease over time.
- Question 2. What is the relationship between *intended use and actual use* of the Medito meditation app?
- Hypothesis 2. Intended use of the Medito app will be associated with actual use (ie, engagement level per primary outcome).
- Question 3. Are there baseline *user factors* that will predict engagement levels over a 30-day period?
- Hypothesis 3. Prospective ratings of readiness to change, expectations for sleep efficacy, expectations for anxiety, expectations for efficacy, and expectations for thriving will predict engagement levels over the 30-day period.

### Deviations and Clarifications

Note that the order of preregistration questions has been adjusted to improve logical flow. In addition, question 1 originally referred to the “Medito mindfulness app,” which has been changed to “Medito meditation app” for clarity and consistency. Question 4 in the preregistration is question 2 in the manuscript, and question 2 is now question 3. “Use” in hypothesis 4 is conceptualized as engagement level as shown by the primary outcome variable. While we initially obtained follow-up data, the number of people who returned follow-up data was relatively small. Therefore, question 3 in the preregistration is not addressed in this manuscript.

## Methods

### Ethical Considerations

#### Ethical Approval and Participant Consent

This study was conducted according to ethical guidelines and was approved by the Office of Research Ethics and Integrity, University of Melbourne (2024-29651-52025-2).

Participants consented to participate in the study via the online Qualtrics survey. Consent information was provided in the online survey, and a plain language statement was available for download. The plain language statement can be found in Multimedia Appendix 1. The consent information included explicitly clarifying the participant’s understanding of their right to withdraw at any time without explanation. Participants consented to secondary analyses. The survey flow prevented anyone who did not provide consent from completing the survey, and all responses included were double-checked for consent provision.

#### Participant Compensation

Our consenting sample before exclusions consisted of 1214 participants. Participants were compensated with one entry into a gift card draw for each survey completed, for 2 possible entries across both surveys. One AUD $250 (US $176.05) gift card was drawn per 250 sequential survey completions, resulting in 4 gift cards for the baseline survey and 1 for the follow-up. This equated to a total prize pool of AUD $1250 (US $825.25).

#### Privacy and Confidentiality

Participant data with identifying information (such as first name, first initial of last name, and email address) were retained for compensation purposes but stored securely according to relevant privacy guidelines and encrypted with Transport Layer Security encryption (also known as HTTPS).

### Study Design and Setting

#### Medito App

Medito is a free meditation app offering guided audio meditations, available on the iOS and Android app stores. Meditators can choose individual sessions or follow structured programs, such as a 30-day introductory challenge.

#### Data Collection

**Figure 1.**
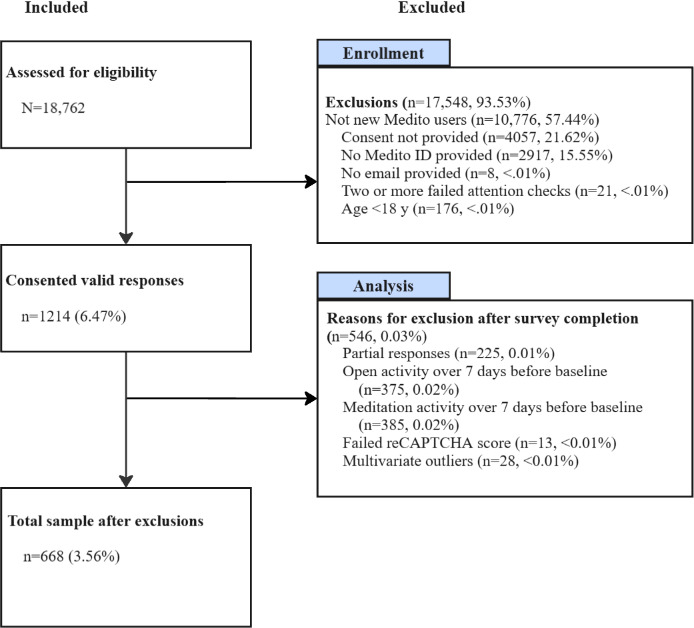
Participant flow diagram*.* The exclusion groups within the enrollment and analysis stages are not mutually exclusive.

The 2 methods of data collection were via a data sharing agreement with the Medito app developers and through a Qualtrics survey. Users provided their Medito ID number when consenting to the research team analyzing their app use. The baseline survey included a screening question, “Did you download Medito for the first time within the past day?” to target new users. The baseline survey was completed at the point of sign-up, and the follow-up survey was distributed for completion 30 days after baseline completion. Participant flow is discussed in the following section and displayed in Figure 1.

### Eligibility and Recruitment

#### Inclusion and Exclusion

Participants had to have downloaded Medito within the previous 24 hours to be eligible. Survey access was restricted to major countries that are predominantly English speaking, along with members of the European Union: Australia, Austria, Belgium, Bulgaria, Canada, Croatia, Cyprus, Czech Republic, Denmark, Estonia, Finland, France, Germany, Greece, Hungary, Ireland, Italy, Latvia, Lithuania, Luxembourg, Malta, the Netherlands, New Zealand, Poland, Portugal, Romania, Spain, Sweden, the United States, and the United Kingdom. These countries were selected due to the predominance of English speakers and approximate financial parity to ensure ethical compensation and consistency in privacy laws.

#### Participant Flow

See Figure 1 and Supplementary 1 in Multimedia Appendix 1 for a detailed description of participant flow.

#### Procedure

Participants were sourced directly from Medito to complete a survey hosted by Qualtrics and advertised on the Medito app platform. The survey was live on the Medito platform from July 20 to August 30, 2024. Follow-up surveys were automatically sent 30 days after baseline completion. Surveys could be completed online using a laptop or mobile device.

### Outcome Variables

#### Engagement Data

Medito provided detailed app usage data, including app opens count, meditation tracks completed, track length, track title, time of activity, meditation tracks started, and streaks (days of consecutive use). Information provided by Medito and used in analysis included Medito IDs for linking data to survey responses, activity time stamps for opens and track listens, and duration of meditation tracks. The total minutes of engagement in the first 30 days were calculated by summing the duration of each track completed in the 30 days following the user’s first app open.

#### Study Outcomes

Total minutes of use in the first 30 days were the primary outcome variable to address research questions 1, 2, and 4. Minutes of use were calculated as a total over the 30-day period following the first time the app was opened by adding the durations of tracks completed. Days of use, meditations completed, and app opens were also collected to provide a holistic view of engagement in addressing question 1. App opens allowed for a comparison between daily app activity in our sample compared with Baumel et al [4]. Intended use patterns were collected to compare with actual use and to address question 4. The follow-up survey addressed the relationship between app factors and engagement outlined in question 3, which will be published elsewhere.

### Data Sources and Measurement

#### Overview

Participants reported their age, gender (eg, male, female, nonbinary, genderfluid, or other), income, and education level. Participants reported the number of apps they had tried, including Medito, ranging from 1 (Medito only) to ≥5. Participants also reported hours of meditation experience in the following ranges: beginner (0‐100 h), intermediate (101‐1000 h), and expert (≥1001 h). Mental health factors included satisfaction with life, meditation-related adverse event impairment level and length, well-being, perceived stress, distress, and quality of life. Expectations were assessed for various domains (eg, sleep, stress, anxiety, attention, happiness, thriving, and performance enhancement), as well as whether the app matched expectations, how confident the user was in improvement, what degree of improvement was expected, and how ready they were to change. Personality factors were based on the Big Five Inventory [55].

#### Meditation-Related Adverse Events Abbreviated 3-item

Part 1 of the Meditation-Related Adverse Events [56], a 2-part scale that assesses meditation-related events, was completed. The first part of the scale consists of 3 items assessing the frequency of perceived adverse effects, the extent of related functional impairment, and the length of functional impairment. Each item has a Likert-style response format, ranging from never to frequently for frequency, not at all to severely for impairment, and 1 day or less to 1 year or longer for length of impairment.

#### The EuroQoL Health and Wellbeing Assessment–Short Form

The EuroQoL [57] assessment is a newly formulated quality of life measure. The scale consists of 9 items on a 5-point scale, where 1 represents “no difficulty,” “none of the time,” or “no physical pain” and 5 represents “unable,” “most or all of the time,” or “very severe physical pain,” depending on the context. In this study, the scale had good internal consistency (α=.86; ω=0.72).

#### Perceived Stress Scale

The Perceived Stress Scale [58] is a brief measure of subjective stress. It ranges from 0 (never) to 4 (almost always). In this study, the scale had reasonable internal consistency (α=.71; ω=0.69).

#### The Kessler Psychological Distress Scale

The Kessler Psychological Distress Scale (K10) [59] was used to assess psychological distress in the last 30 days. The scale consists of 10 items assessing anxiety and depressive symptoms on a 5-point scale, ranging from 1 (none of the time) to 5 (all of the time). In this study, the scale had excellent consistency (α=.91; ω=0.78).

#### The Short Warwick-Edinburgh Mental Wellbeing Scale

The Short Warwick-Edinburgh Mental Wellbeing Scale [60] was used to assess positive elements of mental health and well-being. This scale consists of 7 items on a scale of 1 to 5, ranging from 1 (none of the time) to 5 (all of the time). In this study, the scale showed good consistency (α=.85; ω=0.75)*.*

#### The Satisfaction With Life Survey (1-item)

The Satisfaction With Life Survey [61] Single Item is a 1-item version of the more established Satisfaction with Life Survey. The scale has reasonable criterion validity with the full Satisfaction With Life Survey (zero-order *r*=0.62‐0.64; disattenuated *r*=0.78‐0.80 [61]). The single-item measure asks participants to rate life satisfaction from 1 (extremely dissatisfied) to 7 (extremely satisfied).

#### Expectancy for Various Domains

Medito was rated in general for how effective the user thought it was for the following: anxiety, attention or focus, happiness, thriving, and performance enhancement. The rating scale ranged from 1 (not at all effective) to 7 (extremely effective).

#### Readiness to Change 1-item

The Readiness to Change 1-item [62] assessment is a 10-point scale that assesses an individual’s preparedness to enact a behavioral change. The scale ranges from 0 (not prepared to change) to 10 (already changing). It is based on the transtheoretical model of behavior change stages [63,64]. The readiness ruler captures a construct related to actual readiness [63,65].

#### The Big Five Inventory Short Form 2

The Big Five Inventory Short Form 2 is a 30-item [55] questionnaire that assesses 5 domains of personality as subscales: extraversion, agreeableness, conscientiousness, negative emotionality, and open-mindedness. Each subscale contains 6 items. The subscales representing various personality domains were assessed for internal consistency using Cronbach α and McDonald ω. Cronbach α and ω consistency ranged from poor to excellent (extraversion: ω=0.57; α=.74; agreeableness: ω=0.56, α=.67; conscientiousness: ω=0.59, α=.73; neuroticism: ω=0.69, α=.85; and openness: ω=0.44, α=.63).

### Statistical Analysis

The target sample size was 1000 participants at baseline, providing 90% power to detect a small effect size of *r*=0.102 at α=.05 [14]. The effect size of *r*=0.102 was hypothesized based on prior meta-analyses effect size estimates. The target sample size was determined using G*Power (Heinrich Heine University Düsseldorf) software [66]. Due to unexpected exclusions based on preexisting app use (~30%‐32% of the eligible sample), the final baseline sample size was 668 participants. With this reduced sample, the study retained 90% power to detect an effect of *r*=0.177.

Intended use was individually reported by users across sessions per day, minutes per session, and days per week. The indicated levels of intended use per person were used to calculate the intended total minutes of use over the first 30 days.

A paired-samples 2-tailed *t* test was conducted to compare intended use and actual use (in minutes). Zero-order correlations were calculated to investigate the overall relationship between the factor and the dependent variable. Semipartial correlations were calculated to investigate the unique relationship between a given factor and engagement, controlling for other factors. A regression model was used to investigate which factors showed significant associations with engagement, accounting for multiple predictors. The main outcome variable was total minutes of use over the first 30 days of app use. All factors that approached a significant zero-order association with engagement (*P*<.10) were included in the regression model.

A principal component analysis (PCA) was used to address multicollinearity among predictors and reduce dimensionality in our data. The PCA identified single components within the mental health and expectation outcomes. These were used in additional regression models to further investigate contributions among correlated predictors.

## Results

### Participant Characteristics: Descriptives

Participants ranged from age 18 to 74 (mean 32.2, SD 11.99) years and were close to evenly split between male (n=322, 48.20%) and female (n=318, 47.60%) participants. A small proportion reported nonbinary, gender fluid, or other gender (n=28, 4.20%). Most participants lived in the European Union (n=240, 35.96%) or the United States (n=239, 35.78%). Approximately two-thirds of our sample had completed education above high school (n=458, 68.59%). Most participants were employed full time (n=310, 46.41%) or part time (n=114, 17.08%). Approximately one-fifth were unemployed (n=123, 18.41%). Income was adjusted within countries into 5 brackets, with each bracket covering approximately 20% of the income range for that country. People were evenly distributed across the 4 lower income brackets adjusted for income levels across countries, with fewer (n=77, 11.53%) people in the top income bracket for their country. Notably, participants reported relatively high levels of perceived stress [67] and moderate-to-high psychological distress levels [68] compared to normative English and Australian samples (Table 1).

**Table 1.** Meditation experience and mental health descriptives.

Categories	Values
Meditation experience, n (%)	
Apps tried	
1 (Medito only)	209 (31.25)
2	183 (27.49)
3	171 (25.67)
4	53 (7.93)
No response	52 (77.90)
Hours of meditation experience	
0‐100	545 (81.66)
101‐1000	114 (17.03)
1000+	9 (1.35)
Mental health, mean (SD)	
Perceived stress (PSS-4^a^)	7.20 (3.11)
Psychological distress (K10^b^)	23.87 (8.29)

a PSS-4: Perceived Stress Scale-4.

b K10: 10-item Kessler Psychological Distress Scale.

### Statistical Recording Issues

From July 20 to August 13, Medito indicated a technical anomaly in the recording of statistics for tracks completed and streaks. However, analyses indicated the glitch was unlikely to have seriously affected the data (Supplementary 2 and Supplementary 3 in Multimedia Appendix 1).

### Overall Engagement

**Figure 2.**
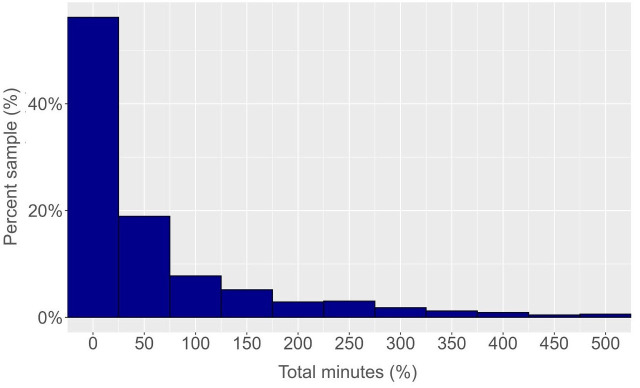
Engagement pattern—total minutes of use in the first 30 days, expressed as a percentage of the sample*.* This figure is truncated for visualization, excluding 21 outliers with values above 1000 min.

**Figure 3.**
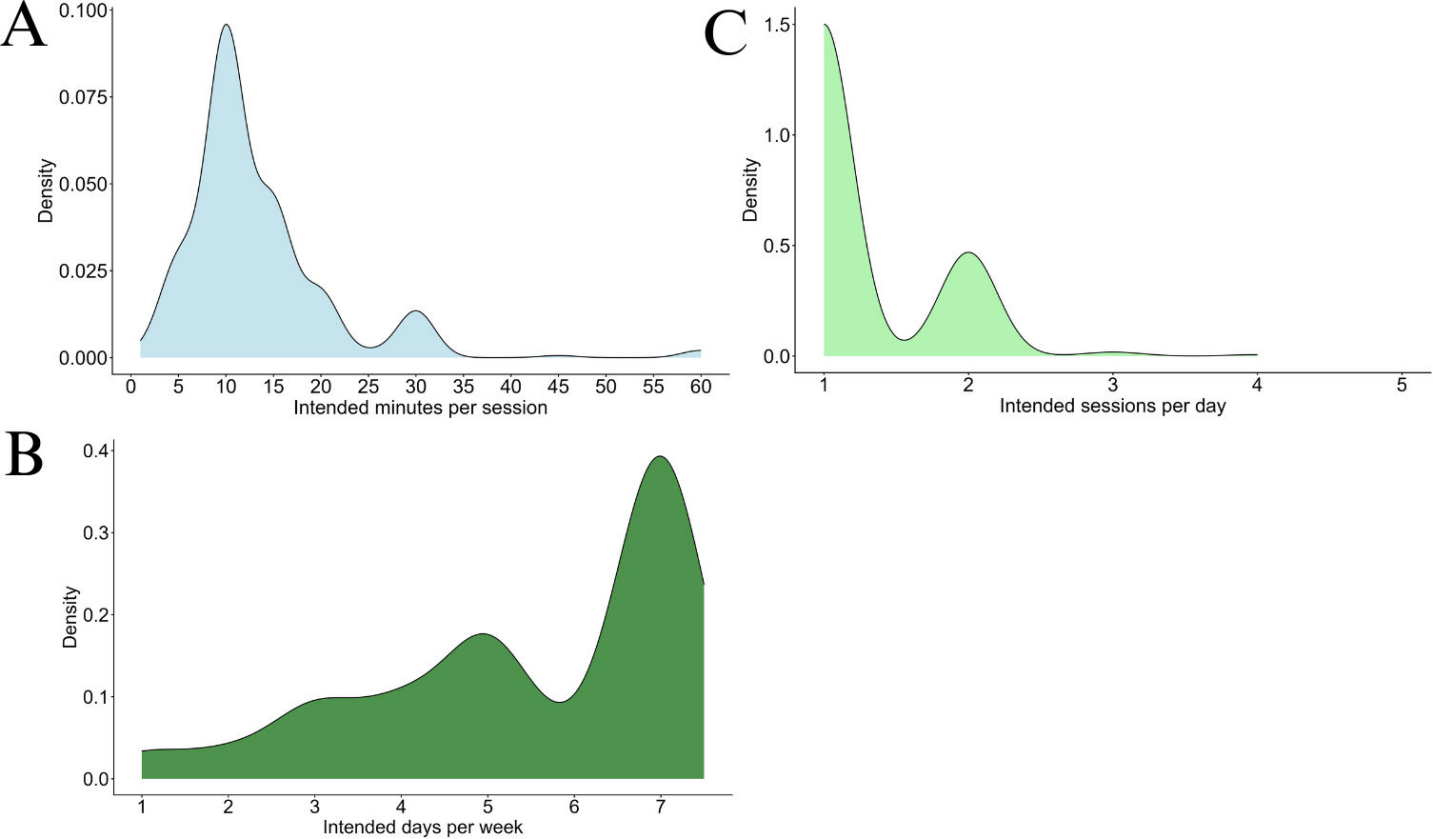
Smoothed density plots showing intended use. Panel A shows intended minutes per session, panel B shows sessions per day, and panel C shows days per week. Panels A to C show intended use by Medito users at the time of download, reported in sessions per day, minutes per session, and days per week. Participants reported intended sessions per day on a scale of 1 to 5 and intended minutes per session from 0 to 60 in approximately 5-min increments (excluding between 30 and 45 min). Intended days per week ranged from 1 to 7 d. Most users (>50%) reported intending to use the app for one 10-min session daily.

To research app engagement, we assessed user engagement levels via total minutes. We further clarified patterns of use by assessing weekly minutes and days of use (Table 2). Engagement was highly positively skewed (mean 70, SD 176.81; median 16.11, IQR 0-74.51 during the first 30 days), suggesting that most participants had low engagement. Most sessions lasted 10 to 11 minutes; approximately half of the users in our sample did not engage beyond approximately one guided meditation. The majority (520/655, 79.4%) of participants engaged for less than 100 minutes in the first 30 days of use (Figure 2 and Table 3), despite the majority of participants intending to engage for 10 minutes per day over the 30-day period (Figures3  4). Engagement dropped quickly over time; on day 7, only 27.68% (n=181) of participants used the app. Approximately one-fifth (n=124, 18.86%) were still engaged on day 14, while 16.26% (n=107) were still engaged at day 30. Overall, a higher proportion of users were engaged each day than in the sample of Baumel et al [4], with a median daily use of 17.47%, compared with 4.10% (see Figure 5 for comparison). No differences in engagement were found by age, sex, education, religion, income, or country (Figures3  6 and Supplementary 4 and Supplementary 5 in Multimedia Appendix 1).

**Table 2.** App use descriptives*.*

Engagement variable	Values, n (%)	Values, mean (SD)	Percentile				
			5%	25%	50%, median (IQR)	75%	95%
Total minutes	655 (98.05)	70.02 (176.81)	0.00	0.00	16.11 (0-74.51)	74.51	292.49
Total sessions completed	668 (100)	10.29 (11.12)	1.00	2.00	6.00 (2-14)	14	34
Tracks started	518 (77.54)	12.48 (14.96)	1.00	3.00	8.00 (3-19)	19	45.15
Duration (d)^a^	455 (68.11)	13.80 (11.80)	0.01	1.43	11.96 (1.43-27)	27	29.75
Minutes per session^b^	468 (70.06)	11.34 (11.57)	2.68	5.01	10.00 (5.01-11.75)	11.75	29.95

a Duration in days equals the number of days between first opening the app and the most recent use within the 30-day period.

b Minutes per session were calculated by taking the statistic for minutes and dividing it by sessions completed for the mean and percentile ranges.

**Table 3.** Sociodemographic features.

Categories	Values
Age (y), mean (SD)	32.20 (11.99)
Gender, n (%)	
Male	318 (47.60)
Female	322 (48.20)
Nonbinary, genderfluid, or other	28 28 (4.20)
Income level^a^, n (%)	
1	159 (23.80)
2	161 (24.10)
3	106 (15.87)
4	77 (11.53)
5	132 (19.76)
Country/continent^b^, n (%)	
European Union	240 (35.96)
United States	239 (35.78)
United Kingdom	53 (7.93)
Australia	57 (8.53)
Canada	64 (9.58)
New Zealand	15 (2.24)
Occupation, n (%)	
Employed part-time	26 (3.89)
Employed full-time	56 (8.38)
Due to start a job within the next month	67 (10.02)
Not in paid work (eg, homemaker, retired, or disabled)	95 (14.22)
Unemployed (and job seeking)	114 (17.07)
Other	310 (46.41)
Religion, n (%)	
No religion	8 (1.20)
Christian	10 (1.50)
Hindu	11 (1.65)
Jewish	14 (2.10)
Muslim	21 (3.14)
Buddhist	47 (7.04)
Sikh	53 (7.93)
Spiritual but not religious	121 (18.11)
Atheist	152 (22.75)
Agnostic	231 (34.58)

a Income level is reported in quintiles standardized for local currency and income distribution, with 1 representing the lowest quintile and 5 the highest. Frequency in each quintile represents the distribution of participants across income brackets.

b See Supplementary Material 5 for full country of residence details. European Union countries have been reported as a frequency together here for brevity.

**Figure 4.**
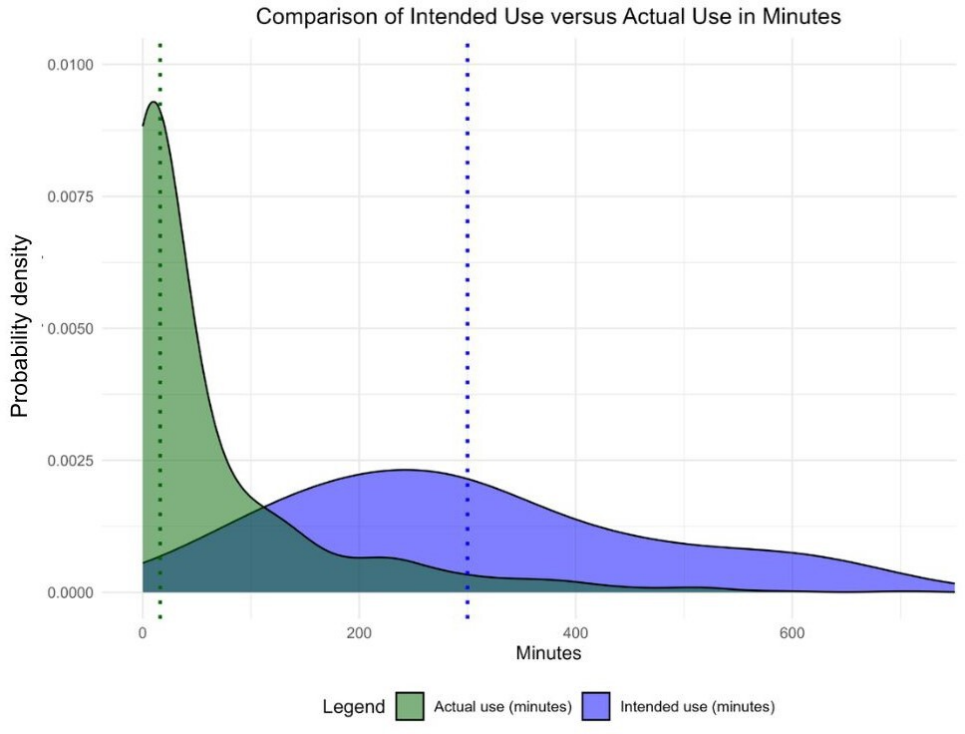
Density curves showing intended and actual minutes of use within the first 30 days, with minutes on the x-axis and probability density on the y-axis. The blue dotted line represents the median intended minutes, and the green dotted line represents the median actual minutes.

**Figure 5.**
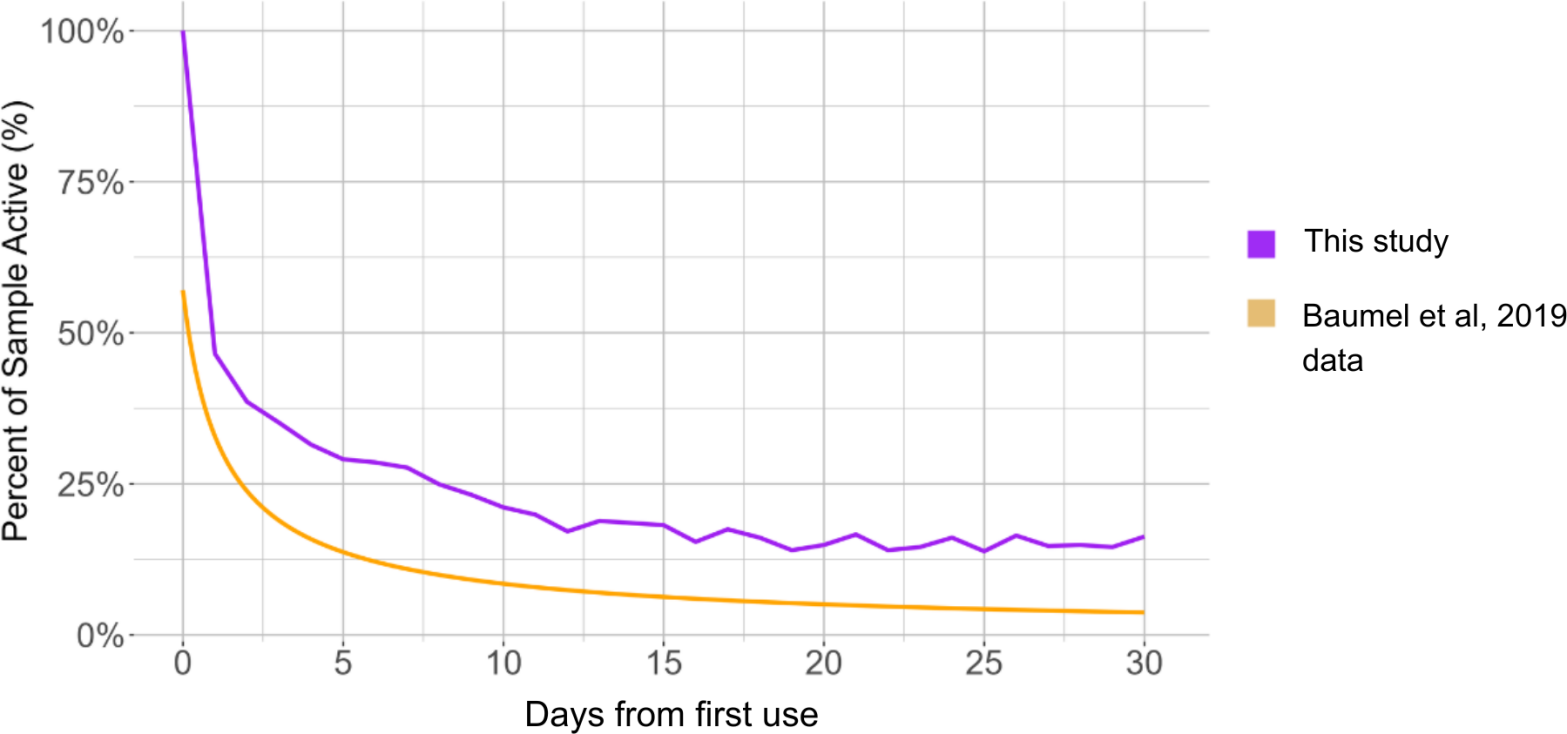
Curve showing the percentage of the sample actively using the app each day after download. App use on a given day represents the percentage of the sample who used the app on that day. The percentage of the sample active per day was used to provide a direct comparison with the findings of Baumel et al [4]. The yellow line represents an estimated curve from Baumel et al [4] and does not reflect the variation in app opens on each given day. The purple line represents the trend line of active use each day in the first 30 days of use in this study.

**Figure 6.**
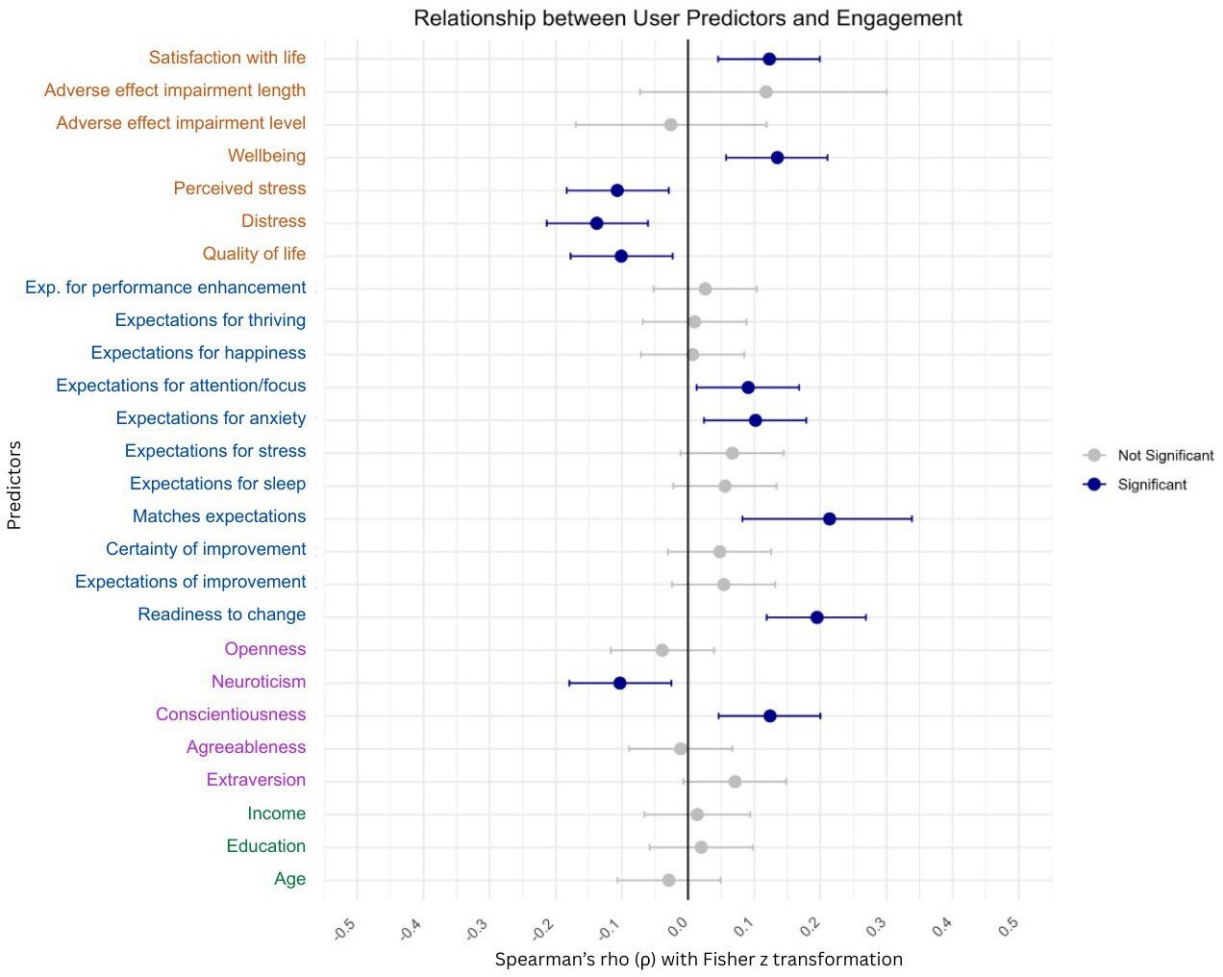
Spearman ρ coefficients of association between predictor factors and total minutes of use over the first 30 days*.* Whiskers represent 95% CI generated using Fisher z transformation. Dark blue represents significant association at *P*<.05; gray represents nonsignificant associations. Orange represents mental health factors, dark blue represents expectations factors, purple represents personality factors, and green represents sociodemographic factors. Quality of life is reverse coded; therefore, a negative association indicates that higher quality of life relates to higher engagement.

### Patterns of Engagement

Figure 5 shows the percentage of participants actively using the app each day following download.

### Intended Versus Actual Use

Figure 3 illustrates participants’ intended app use at the time of download across 3 dimensions: minutes per session, sessions per day, and days per week.

### Intended Use in Comparison With Actual Use

To address question 2, we investigated the relationship between intended and actual use. There was a weak associative relationship (*r*=0.094; *P*=.02). Figure 4 shows the density curve and median intended use stated as baseline (purple) compared with actual use over the first 30 days (green). Users intended a median of 300 (IQR 192.86-482.14) minutes over the first 30 days but only engaged for a median of 16 (IQR 0-74.51) minutes. In other words, median intended use was nearly 19 times greater than actual use. This suggests that users who intended to meditate more were more likely to do so (ρ=0.106; *P*=.007).

However, as expected, there was a large difference whereby intended use was significantly higher than actual use (mean difference 350.61, SD 493.53, 95% CI 312.72-388.51 min; Cohen *d*=0.710; *t*_653_=18.168; *P*<.001; Figure 4).

### Predictors of Engagement: User Factors (Question 2, Hypothesis 2)

Zero-order correlations for user factors are displayed in the forest plot below (Figure 6). Zero-order correlations, semipartial correlations, and regression beta weights are reported in Table 4.

**Table 4.** Zero-order correlation, semipartial correlations, and regression beta weights for predictors*.* * <.05, ** <.01 and *** <.001

Variable	ρ	sr^a^	β^b^
Mental health factors			
Satisfaction with life (SWLS^c^)	0.123**	0.038	.008
Well-being (WEMWBS^d^)	0.135**^,^***	0.020	.010
Perceived stress (PSS-4^e^)	−0.107**	0.027	.020
Psychological distress (K10^f^)	−0.138***	−0.070	−0.022
Quality of life (EQ-HWB-S^g^)	−0.10*	0.037	<.001
Expectation factors			
Expectations for sleep	0.056	0.026	.001
Expectations for stress	0.067	−0.050	−0.012
Expectations for anxiety	0.102*	0.063	.014
Expectations for attention/focus	0.091*	0.014	.003
Personality factors			
BFI2^h^—conscientiousness	0.124**	0.055	.002
BFI2—neuroticism	−0.103*	0.033	.010
Readiness to change	0.195***	0.146	.012

a sr: semipartial correlation.

b *R*2=0.041.

c SWLS: Satisfaction with Life Survey.

d WEMWBS: Warwick-Edinburgh Mental Wellbeing Scale.

e PSS-4: Perceived Stress Scale-4.

f K10: 10-item Kessler Psychological Distress Scale.

g EQ-HWB-S: EuroQol Health and Wellbeing Scale.

h BFI2: Big Five Inventory Short Form 2 (30-item).

### Mental Health Factors

All mental health factors were negatively associated with engagement in the zero-order correlations, indicating that poorer mental health, regardless of scale direction, was linked to lower engagement (Table 4). Only psychological distress showed a negative semipartial relationship with engagement that approached significance (sr=−0.070; *P*=.09). Perceived stress was the only significant mental health factor in the multiple regression, showing a positive association to engagement (β=.02; *P*=.04), contrasting with its zero-order correlation.

### User Factors

Readiness to change showed the largest zero-order and semipartial correlation (ρ=0.194, *P*<.001; and sr=0.156; *P*<.001). Of the personality factors, conscientiousness and neuroticism (positive relationship) and neuroticism (negative relationship) correlated with engagement (ρ=0.125, *P*=.002; and ρ=−0.102, *P*=.009). Expectations for anxiety (ρ=0.102; *P*=.01) and attention (ρ=0.091; *P*=.02) were weakly positively associated with engagement. No user factors were significant in the regression model.

### Regression Models With PCA Components

Multicollinearity was observed within the regression (Figure S2 in Multimedia Appendix 1). As a result, we undertook PCAs for mental health and expectations factors. Both mental health and expectations each showed one central factor with high loadings from contributing factors that accounted for a majority of the variance (Tables S9-S12 in Multimedia Appendix 1). Regression models were conducted using the mental health and expectations components found in the PCA (Tables S6-S8 in Multimedia Appendix 1).

With both components included, no factor or component achieved significance, and the amount of variance was reduced substantially (*R^2^*=0.02; Table S7 in Multimedia Appendix 1). In a regression containing the expectations component, only perceived stress was significant (β=.022; *P*=.03), and the expectations component was nonsignificant, in line with prior results (Table S8 in Multimedia Appendix 1). In a regression containing the mental health component, the mental health component (β=−0.008; *P*=.049), readiness to change (*β*=.011; *P*=.048), and expectations for anxiety (β=.015; *P*=.049) were significant (Table 5). Deviating from the main model, readiness to change and expectations for anxiety were significant positive predictors when controlling for overlap among measures of mental health. Furthermore, the mental health component, representing mental ill health, also negatively predicted engagement. The negative relationship between mental ill health and engagement suggests that worse mental health results in lower engagement.

**Table 5.** Regression model with mental health component only. *<.05 and *** <.001

Variable	Standardized estimate^a^^,^^b^	SE	*t* value	Pr (>|t|)
Intercept	−0.313***	0.012	−26.832	<0.001
User factors				
Mental health component	−0.008*	0.003	−1.976	0.049
Conscientiousness (BFI)^c^	0.001	0.006	0.241	0.81
Neuroticism (BFI)	0.008	0.008	0.998	0.32
Readiness to change	0.011*	0.006	1.983	0.048
Expectations for sleep	0.001	0.008	0.137	0.89
Expectations for stress	−0.013	0.009	−1.441	0.15
Expectations for anxiety	0.015*	0.008	1.976	0.049
Expectations for attention/focus	0.002	0.007	0.352	0.73

a *R*2=0.027.

b *R*=0.015.

c BFI: Big Five Inventory.

## Discussion

### Principal Findings

This study investigates low rates of meditation app engagement in prior literature by examining prospective predictors of use. Minutes of use in the first 30 days after download were compared with user characteristics among 668 participants. While our findings align with prior works [4], our data showed a more gradual decline in use than has previously been observed. Actual use departed from intended use, with participants meditating about 350 minutes less (on average) than planned. Most participants aimed to meditate for 10 minutes daily but only engaged in approximately one 10-minute session. Nevertheless, there was a correlation between intended and actual use, suggesting that those who intended to meditate more practiced slightly more. The gap between intended and actual meditation time reinforces prior findings regarding the intention-behavior gap and engagement challenges with digital interventions [4,19-21,69].

Contrary to hypotheses, perceived stress was the only significant predictor of engagement in the regression model. Expectations for anxiety (positive) and readiness to change (positive) reached significance when modeling mental health as a single component. Higher stress may motivate behavioral change, reinforced by beliefs in meditation’s efficacy for anxiety to manage acute stress. Our findings suggest that baseline characteristics, including stress, expectations, and readiness to change, predict initial meditation app use, implicating these factors in habit-building as well as drop-off.

### Mental Health

As expected, measures of mental ill health, such as distress and perceived stress, were associated with lower engagement, while measures of positive mental health, such as satisfaction with life and well-being, were associated with greater engagement. Depression is related to low adherence to, and attrition from, meditation and health interventions [54]. This may reflect a broader message about the ability for people experiencing mental ill health to engage in meditation apps. People with significant psychological distress and psychoticism may disengage prematurely due to the increased likelihood of meditation-related adverse events [70]. Mental ill health also often reduces executive functioning, cognitive functioning, and affective functioning, which can interfere with one’s ability to set goals or follow through on health-related goals or behaviors [71]. A reduction from normal functioning is itself a key criterion for many psychological disorders. This is not a new issue for engagement in mental health interventions but underscores that mental health difficulties hinder the meaningful engagement required to experience change. Having good mental well-being, on the other hand, may relate to greater energy, motivation, and self-efficacy when making behavior change, all of which are either further promoted and reinforced by consistent meditation app use. People scoring higher on well-being and satisfaction may also face less difficulty with the act of meditation, as they may have less difficulty dealing with negative thoughts or experience fewer negative thoughts overall [53].

While not mental health per se, consistent with this pattern, lower quality of life is associated with lower engagement. This means health issues in general may mean more difficulty in engaging, although barriers to health care motivate initial engagement [33]. Low quality of life may result in a lower capacity to self-regulate, plan, or cope with challenges [72].

The relationship between perceived stress shifted direction from negative (on its own) to positive (controlling for other variables), suggesting that there may be a unique contribution of acute stress unrelated to mental health. The perceived stress scale potentially reflects different latent variables when presented on its own (acute stress) versus as part of a mental health component variable (chronic stress) [73]; therefore, controlling for mental health may remove shared variance related to chronic stress, leaving the remaining variance to reflect more acute experiences. While mental health and chronic stress may present a barrier to engagement [54,74], our most robust finding shows that higher perceived stress drives higher engagement. Meditation apps have shown preliminary efficacy for perceived stress [75], and population-level work shows that people may seek out meditation to address unmet needs in mental health [33].

Our nonclinical population reported high levels of perceived stress and psychological distress relative to normative samples. Individuals may have downloaded the meditation app as a step toward alleviating their distress but may have found it harder to engage with significant mental ill health or chronic stress, reflected in the negative relationship between the mental health component and engagement. Given the moderate effects of meditation apps on perceived stress [14], some level of perceived relief may be felt early, promoting further engagement. Preliminary evidence suggests one session of meditation may be enough to produce at least a temporary reduction in perceived stress [76].

### Readiness and Expectations

Readiness to change accounted for the most unique variance and approached significance in the regression model, showing the strongest zero-order correlation with engagement. However, robust regression downweights outliers, which contribute to discrepancies between semipartial correlations and regression beta weights, may have reduced the contributions of readiness to change in the regression model (Table 5). Readiness was no longer significantly associated when controlling for other factors but significant with a single mental health component. When mental ill health and/or poor well-being were modeled as one component, variance potentially capturing desire to address life dissatisfaction and/or acute stress might then best be captured by readiness to change. The meaningful positive association of readiness to change is consistent with prior work showing that readiness to change is a central facet of behavior change [7,65] and that intrinsic motivation predicts subsequent behavior [32], and that intrinsic motivation predicts subsequent behavior [32].

Out of the expectations domains, only expectations for anxiety accounted for substantial unique variance and remained significant when modeling one mental health component. This may indicate that alleviating anxiety motivates app-based meditation and aligns with mental health as a common motivator for commencing meditation [6]. While it could not be included in the main regression model due to limited responses (n=221), expectation match showed the strongest correlation with engagement. This indicates that while expectations are important, users may discontinue practice if benefits are not observed or anxiety is potentially exacerbated [77], as app-based meditation shows smaller effect sizes for anxiety than depression or perceived stress [14,77]. In other words, expectations interact with experience to influence behavior.

### Personality

Conscientiousness and neuroticism were both significantly associated with engagement but were not significant in the regression model. This is in contrast to prior work that suggests agreeableness, neuroticism, and openness are related to meditation engagement [43]. It is logical that conscientiousness would predict higher engagement, given that conscientious traits are focused on self-discipline [55]. Previously, longitudinal studies found that higher baseline conscientiousness predicts higher adherence to meditation, which in turn predicts higher stress reduction [78]. However, one might also logically expect openness to be important in trying a new habit. It may be that since buy-in for meditation apps is much lower than, for example, signing up for an extended in-person course, openness matters less than simply the diligence of continuing (ie, conscientiousness).

Neuroticism negatively predicting engagement is consistent with our finding that mental health factors are negatively associated with engagement and the mental health component negatively predicts engagement in the regression. Higher neuroticism, or the tendency to experience negative emotion, relates to a higher perception of barriers to meditation, likely due to the impact of rumination on self-efficacy, readiness, and the subjective experience of meditation [42,46,79]. Difficult or negative thoughts are common challenges in beginning meditation [48], and people high in neuroticism may face more negative thoughts or perceive these thoughts as more of an obstacle. Sitting with one’s thoughts may be more difficult for those with a more negative predisposition. Neuroticism is distinct from low mood or mental ill health, as it reflects an overall underlying tendency to negativity; however, neuroticism is a strong predictor of mental ill health, as neuroticism exacerbates stress and interferes with coping [80]. However, interventions for mental health can modestly reduce neuroticism [81,82], suggesting that persistence has positive outcomes, although it is important to differentiate between challenges that are part of the learning process and harmful adverse events [56].

While the prior study by Khwaja et al [43] investigated meditation app use via self-report usage logs, and our previous survey used objective app data but retrospective factors, this study uses objective app engagement data with a larger sample, which may be more reliable. Personality may play a smaller role in engagement than perceived stress and readiness to change during relatively short periods of monitoring use.

Overall, people are commencing meditation app use when they believe the app is effective and are cognizant of their stress. The significance of the mental health component, readiness, expectations, and perceived stress suggests a connection between engagement and awareness of mental ill health and/or acute stress, making a conscious decision to address it and believing that the intervention will work.

### Limitations

This study was limited by a reduced sample size with power to detect effects equivalent to *r*=0.177. While it is possible that we were underpowered, it is notable that other meditation and mental health app engagement studies have observed larger effect sizes, suggesting that the true effects are smaller than what has been previously suggested [14,17,37,53]. Generalizability is limited, as the study focused on a single meditation app, although this is a common approach [19,20]. This ensured design and quality consistency but restricts applicability to other apps. Future work could compare engagement levels across apps.

A technical error in the recording of tracks completed occurred during the study period. No significant difference was found in the relationship between completed and started tracks. Nonetheless, the glitch may have introduced additional noise, affecting our ability to detect relationships. The inclusion of started tracks in the engagement variable could have partially accounted for unrecorded tracks, but without knowing the percentage of each track completed, engagement duration would remain unclear.

Our model only accounted for a small amount of variance (*R*^2^=0.041, 4%), compared to 13% to 24% found in other behavior change studies [45,83]. This suggests key factors are missing. However, prior work largely relied on cross-sectional reports or recall, relating more to baseline or intended behavior rather than objective engagement level. Other studies examining out-of-class meditation practice predicted 21% variance [45]. Unconsidered factors may include app preferences, unconsidered demographic differences, psychological traits, or contextual variables. Predicting meditation may also be harder than other health-related behaviors due to less short-term tangible reinforcement, higher perceived effort, and the intentional nature of the behavior as opposed to the habitual or automatic nature of other behavior change. Future research could build on the current and preceding studies to further refine predictors and improve model power.

### Future Directions

Future work could compare 30-day follow-up data with initial findings and detect whether changes over time predict engagement. This could explore differences between expectations and perceptions after initial versus ongoing use. A greater gap between baseline and follow-up ratings might suggest that exceeded expectations may promote engagement, whereas expectations not met or matched may have a lesser influence. Notably, however, steep discontinuation rates may necessitate large sample sizes to make such work possible. If only 10% to 20% of users are still actively engaged after 30 days, samples would need to be substantial to include a large enough proportion of continuing users.

Additionally, follow-up data may allow for mobile app ratings such as the user Mobile Application Rating Scale, which would provide clearer insights into users' app perceptions after 3 days of use. Digital therapeutic alliance or the relationship between the user and the digital intervention [84] and user Mobile Application Rating Scale [85] engagement (ie, appeal) merits further investigation as they have some of the strongest zero-order correlations with engagement [7].

### Conclusions

This study reveals that half of new meditation app users disengage within the first 2 weeks of use, and the majority disengage by the end of 30 days. While most users plan daily engagement, they tend to complete only a single 10-minute session. Perceived stress is the strongest predictor of engagement, while mental health, readiness to change, and expectations for anxiety management show predictive use. While most users disengage, those who are under stress, do not have pronounced mental ill health, are motivated to make changes, and/or have reasonably high expectations that a meditation app can reduce anxiety are more likely to continue using the app. Given the growing role of meditation apps in public mental health [3,4], understanding the motivations and expectations of meditation app users at baseline provides valuable insight into how and why meditation apps are being used in nonclinical populations.

## Supplementary material

10.2196/79366 Multimedia Appendix 1 Supplementary materials that support the main analyses.

## Abbreviations

K10: Kessler Psychological Distress Scale
MBP: mindfulness-based programs
PCA: principal component analysis
