# Supplementary material for: Real-World Meditation App Engagement: Longitudinal Study of the Medito Meditation App
Source: JMIR Mhealth Uhealth. 2026 Jun 8;14:e79366. doi: 10.2196/79366 (PMC13245846; doi:10.2196/79366)
Supplement: Multimedia Appendix 1 [file mhealth-v14-e79366-s001.docx]

**Supplementary Materials 3**

[Supplementary 3.1. Participant Flow description. 1](#_Toc209095985)

[Supplementary 3.2. Statistical recording issue description 2](#_Toc209095986)

[Supplementary 3.3. Regression Models comparing tracks started versus completed during statistical recording error 3](#_Toc209095987)

[Supplementary 3.4. Assessment of Relationship between Sociodemographic Factors and Engagement 3](#_Toc209095988)

[Supplementary 3.5. Correlations between predictor variables and engagement 4](#_Toc209095989)

[Supplementary 3.6. Semi-partial correlations between predictor variables and engagement. 5](#_Toc209095990)

[Supplementary 3.7. Main regression model assessing relationship between predictors and engagement 5](#_Toc209095991)

[Supplementary 3.8. Regression Model with Expectation and Mental Health components. 6](#_Toc209095992)

[Supplementary 3.9. Tests for Normality, Linearity and Multicollinearity 7](#_Toc209095993)

[Supplementary 3.10. Principal Component Analysis (PCA) 9](#_Toc209095994)

[Supplementary 3.11. Scatterplots comparing factor score with engagement level in total minutes (log) for all factors significant in the regression 11](#_Toc209095995)

Supplementary 1. Participant Flow description.

18762 respondents completed the pre-screening question, which asked if the user was a new Medito user prior to the full assessment battery. We excluded 17,406 responses for the following reasons: 10776 were not new Medito users, 4057 did not consent, 2917 provided no Medito ID, 8 provided no email, and 21 failed attention checks, and 176 reported an age below 18 so did not provide informed consent. Some people fell into one or more of these categories. 1214 responses consented, provided their email and Medito ID and commenced the baseline questionnaire. Six were identified as duplicate responses, in which case the most complete response was retained.

Of the baseline responses, we excluded 434 for being non-new users. A sum of 145 had opens activity more than 7 days before baseline, 375 had completed a meditation track more than 7 days before baseline and 385 had started a meditation track more than 7 days before baseline. A total of 7 users with activity between September 5^th^ and 15^th^ were excluded due to a data gap during this period. There were 225 partial responses (less than 100 percent complete). Responses that failed two or more attention checks were excluded. As a result, 37 responses were excluded from analysis. One user was excluded for having a survey completion time below a reasonable duration (<7.5 minutes). ReCAPTCHA scores distinguishing human activity from automated or ‘bot’ activity detected 13 responses with reCAPTCHA below 0.5, indicating likely bots. Of the sample, 28 were identified as multivariate outliers in the 99^th^ percentile and removed. Two participants were removed for being from countries outside the scope. Some participants fell into more than one of the above categories. After these exclusions, the final sample consisted of 668 individuals.

Supplementary 2. Statistical recording issue description

From July 20^th^ to August 13^th^, Medito indicated an anomaly in the recording of statistics for tracks completed and streaks. Data accounting for when a user started a track was unaffected. To examine whether the recording glitch impacted the data, we regressed the percentage of tracks completed (the affected variable) on tracks started (the unaffected variable) with a grouping variable (affected vs unaffected) and an interaction between tracks started and the grouping variable. One might presume that the relationship between tracks started and completed would be affected if the glitch resulted in significantly fewer completed tracks being recorded. While the number of tracks started was a significant predictor of tracks completed, there was no effect of group or its interaction on tracks completed (see Table S2). As these results suggested a consistent, albeit negative relationship between number of tracks started and overall tracks completed with no observable effect of the glitch, we proceeded on the assumption that the glitch merely resulted in random noise among the data.

Supplementary 3. Regression Models comparing tracks started versus completed during statistical recording error

Table S1.

*Regression Model for the relationship between percentage of tracks completed and total tracks started across groups affected and unaffected by statistics recording error.*

|  | Estimate | Standard Error | T value | Pr (>\|t\|) |
| --- | --- | --- | --- | --- |
| Intercept | 82.752 | 5.3626 | 15.431 | <2e-16*** |
| Tracks started | -0.864 | 0.3760 | -2.296 | 0.022* |
| Group (affected/ unaffected) | 7.0123 | 6.1509 | 1.140 | 0.255 |
| Interaction (Tracks started x Group) | -0.353 | 0.434 | -0.814 | 0.416 |

*Note:* *** = p < .001, ** = p < .01, * = p < .05, ^#^ = <.1.

Table S2.

*Regression Model for the relationship between percentage of tracks completed and total tracks started across groups affected and unaffected by statistics recording error (scaled)*

|  | Estimate | Standard Error | T value | Pr (>\|t\|) |
| --- | --- | --- | --- | --- |
| Intercept | -0.509 | 0.055 | -9.337 | <.001*** |
| Tracks started | -0.237 | 0.072 | -3.300 | 0.022* |
| Group (affected/ unaffected) | 0.075 | 0.095 | 0.781 | 0.435 |
| Interaction (Tracks started x Group) | -0.074 | 0.091 | -0.814 | 0.416 |
| Adjusted R^2^ | 0.053 |  |  |  |

*Note:* *** = p < .001, ** = p < .01, * = p < .05, ^#^= <.1.

**Supplementary 4. Country of Residence Descriptives for Full Sample.**

Table S3.

*Country of Residence Descriptives for Full Sample.*

| **Country** | **Total** |
| --- | --- |
| United States | 239 |
| Canada | 64 |
| Australia | 57 |
| Germany | 53 |
| United Kingdom | 53 |
| Italy | 26 |
| Poland | 19 |
| France | 16 |
| New Zealand | 15 |
| Portugal | 15 |
| Spain | 15 |
| Romania | 13 |
| Sweden | 9 |
| Croatia | 8 |
| Hungary | 8 |
| The Netherlands | 8 |
| Austria | 7 |
| Belgium | 7 |
| Greece | 7 |
| Estonia | 6 |
| Czechia | 5 |
| Finland | 5 |
| Ireland | 5 |
| Bulgaria | 3 |
| Lithuania | 3 |
| Latvia | 1 |
| Luxembourg | 1 |

Supplementary 5. Assessment of Relationship between Sociodemographic Factors and Engagement

Table S4.

*Relationship between sociodemographic factors and engagement as total duration in minutes.*

| Variable | Metric | N | *p* |
| --- | --- | --- | --- |
| Sex ^a^ | 52194 | 655 | 0.282 |
| Religion ^b^ | <0.001 | 655 | 0.9919 |
| Country of Residence ^b^ | 0.385 | 655 | 0.9989 |

Note: a = Wilcox rank sum test with continuity correction, b = F value from Type III Anova.

Supplementary 6. Correlations between predictor variables and engagement

Table S5.

*Correlations between predictor variables and engagement as total duration in minutes.*

| Variable | Corr. Coefficient | N | *p* |
| --- | --- | --- | --- |
| ***Demographic Factors*** |  |  |  |
| Age | -0.029 | 655 | 0.461 |
| Education | 0.013 | 655 | 0.610 |
| Income | 0.01 | 623 | 0.735 |
| ***User Factors*** |  |  |  |
| Satisfaction with Life | 0.123 | 655 | 0.002** |
| Wellbeing | 0.135 | 655 | <.001*** |
| Perceived Stress | -0.107 | 655 | 0.007* |
| Distress | -0.138 | 655 | <.001*** |
| Quality of Life | -0.100 | 654 | 0.011* |
| Adverse Impairment Level | -0.020 | 197 | 0.730 |
| Adverse Impairment Length | 0.118 | 119 | 0.225 |
| Expectations for Sleep | 0.056 | 653 | 0.16 |
| Expectations for Stress | 0.067 | 653 | 0.~~08~~092 |
| Expectations for Anxiety | 0.102 | 653 | 0.010* |
| Expectations for Attention/Focus | 0.091 | 653 | 0.023* |
| Expectations for Happiness | 0.007 | 653 | 0.852 |
| Expectations for Thriving | 0.011 | 653 | 0.80 |
| Expectations for Performance Enhancement | 0.026 | 653 | 0.510 |
| Expectation Match | 0.214 | 222 | 0.002** |
| Expectations of Improvement | 0.054 | 653 | 0.172 |
| Certainty of Improvement | 0.048 | 653 | 0.232 |
| Openness | -0.039 | 655 | 0.330 |
| Conscientiousness | 0.124 | 655 | 0.002** |
| Agreeableness | -0.011 | 655 | 0.778 |
| Neuroticism | -0.103 | 655 | 0.010* |
| Extraversion | 0.071 | 655 | 0.074 |
| Readiness to Change | 0.195 | 655 | <.001*** |

*Note:* *** = p < .001, ** = p < .01, * = p < .05, ^#^= <.1.

Supplementary 7. Semi-partial correlations between predictor variables and engagement.

Table S6.

*Matched sample size with regression and semi-partial correlation.*

| Variable | Corr. Coefficient | N | P |
| --- | --- | --- | --- |
| ***User Factors*** |  |  |  |
| Satisfaction with Life | 0.038 | 652 | 0.341 |
| Wellbeing | 0.020 | 652 | 0.606 |
| Perceived Stress | 0.027 | 652 | 0.499 |
| Distress | 0.070 | 652 | 0.075 |
| Quality of Life | 0.037 | 652 | 0.344 |
| Expectations for Sleep | 0.026 | 652 | 0.513 |
| Expectations for Stress | -0.050 | 652 | 0.208 |
| Expectations for Anxiety | 0.063 | 652 | 0.109 |
| Expectations for Attention/Focus | 0.014 | 652 | 0.731 |
| Conscientiousness | 0.055 | 652 | 0.167 |
| Neuroticism | 0.040 | 652 | 0.391 |
| Readiness to Change | 0.146 | 652 | <.001*** |

*Note:* *** = p < .001, ** = p < .01, * = p < .05, ^#^= <.1.

Supplementary 8. Main regression model assessing relationship between predictors and engagement

Table S7.

*Regression Model.*

| Variable | Estimate | Standard Error | T value | Pr (>\|t\|) |
| --- | --- | --- | --- | --- |
| (Intercept) | -0.311 | 0.012 | -26.026 | <.001*** |
| ***User Factors*** |  |  |  |  |
| Satisfaction with Life | 0.008 | 0.012 | 0.912 | 0.362 |
| Wellbeing | 0.010 | 0.008 | 1.162 | 0.246 |
| Perceived Stress | 0.020* | 0.009 | 2.027 | 0.043 |
| Distress | -0.022^#^ | 0.010 | -1.907 | 0.057 |
| Quality of Life | <.001 | 0.010 | 0.060 | 0.952 |
| Expectations for Sleep | 0.001 | 0.008 | 0.191 | 0.849 |
| Expectations for Stress | -0.012 | 0.009 | -1.298 | 0.195 |
| Expectations for Anxiety | 0.014^#^ | 0.008 | 1.717 | 0.086^#^ |
| Expectations for Attention/Focus | 0.003 | 0.007 | 0.498 | 0.618 |
| Conscientiousness | 0.002 | 0.006 | 0.312 | 0.755 |
| Neuroticism | 0.010 | 0.009 | 1.169 | 0.242 |
| Readiness to Change | 0.012^#^ | 0.006 | 1.953 | 0.051^#^ |
| *R^2^* | 0.023 |  |  |  |

*Note:* *** = p < .001, ** = p < .01, * = p < .05, ^#^ = <.1.

Across the two regression models, only perceived stress accounted for significant variation. The models explained little of the variation in engagement.

Supplementary 9. Regression Model with Expectation and Mental Health components.

Table S8.

*Regression Model with Expectation and Mental Health components.*

|  | Estimate | Standard Error | T value | Pr (>\|t\|) |
| --- | --- | --- | --- | --- |
| Intercept | 0.314 | 0.012 | -26.882 | <0.001*** |
| ***User Factors*** |  |  |  |  |
| Expectations Component | -0.003 | 0.003 | -0.803 | 0.422 |
| Mental Health Component | -0.007 | 0.004 | 1.898 | 0.058^#^ |
| Conscientiousness (BFI) | 0.002 | 0.006 | 0.349 | 0.727 |
| Neuroticism (BFI) | 0.008 | 0.008 | 1.060 | 0.290 |
| Readiness to Change | 0.011 | 0.006 | 1.889 | 0.059^#^ |
| *R^2^* | 0.020 | R = 0.013 |  |  |

*Note:* *** = p < .001, ** = p < .01, * = p < .05, ^#^ = <.1.

Table S9.

*Regression Model with Expectation component only.*

|  | Estimate | Standard Error | T value | Pr (>\|t\|) |
| --- | --- | --- | --- | --- |
| Intercept | -0.312 | 0.012 | -26.135 | <0.001*** |
| ***User Factors*** |  |  |  |  |
| Expectations Component | 0.003 | 0.003 | 0.922 | 0.357 |
| Conscientiousness (BFI) | 0.003 | 0.006 | 0.415 | 0.678 |
| Neuroticism (BFI) | 0.010 | 0.008 | 1.201 | 0.230 |
| Readiness to Change | 0.012 | 0.006 | 1.895 | 0.058^#^ |
| Satisfaction with Life | 0.008 | 0.008 | 0.932 | 0.352 |
| Wellbeing | 0.010 | 0.008 | 1.188 | 0.235 |
| Perceived Stress | 0.022 | 0.010 | 2.207 | 0.027* |
| Distress | -0.021 | 0.011 | -1.912 | 0.056^#^ |
| Quality of Life | <-0.001 | 0.009 | -0.024 | 0.981 |
| *R^2^* | 0.035 | R = 0.021 |  |  |

*Note:* *** = p < .001, ** = p < .01, * = p < .05, ^#^ = <.1.

Supplementary 10. Tests for Normality, Linearity and Multicollinearity

**Linearity assumption tests**

**RESET Test**

RESET = 0.91, df1 = 2, df2 = 206, *p* = 0.405

The p-value is greater than 0.05, suggesting no significant evidence of misspecification in the model via fitted values.

**Shapiro-Wilk Normality Test**

W = 0.374, *p* < . 001

Significant p-value indicates residuals deviate from normality.

**Breusch-Pagan Test:**

BP = 11.15, df = 12, *p* = 0.516

Non-significant p-value indicates homoscedasticity.

**Variance Inflation Factor**

Maximum VIF = 6.09 (Perceived Stress)

VIF exceeds 5, indicating potential multicollinearity.


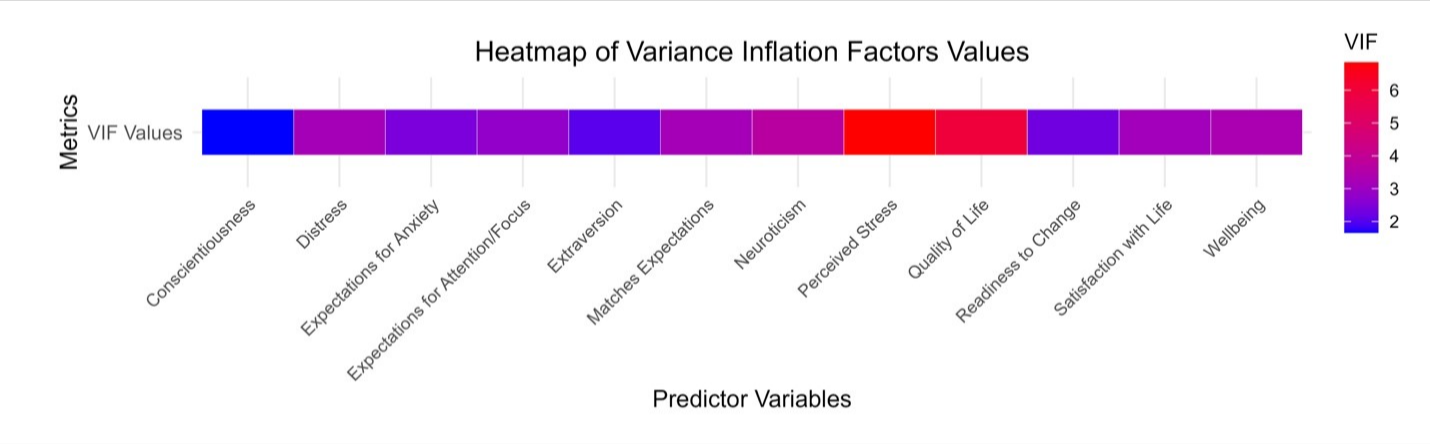


Figure S1.

*Heat map of Variance Inflation Factor Values (VIF).*


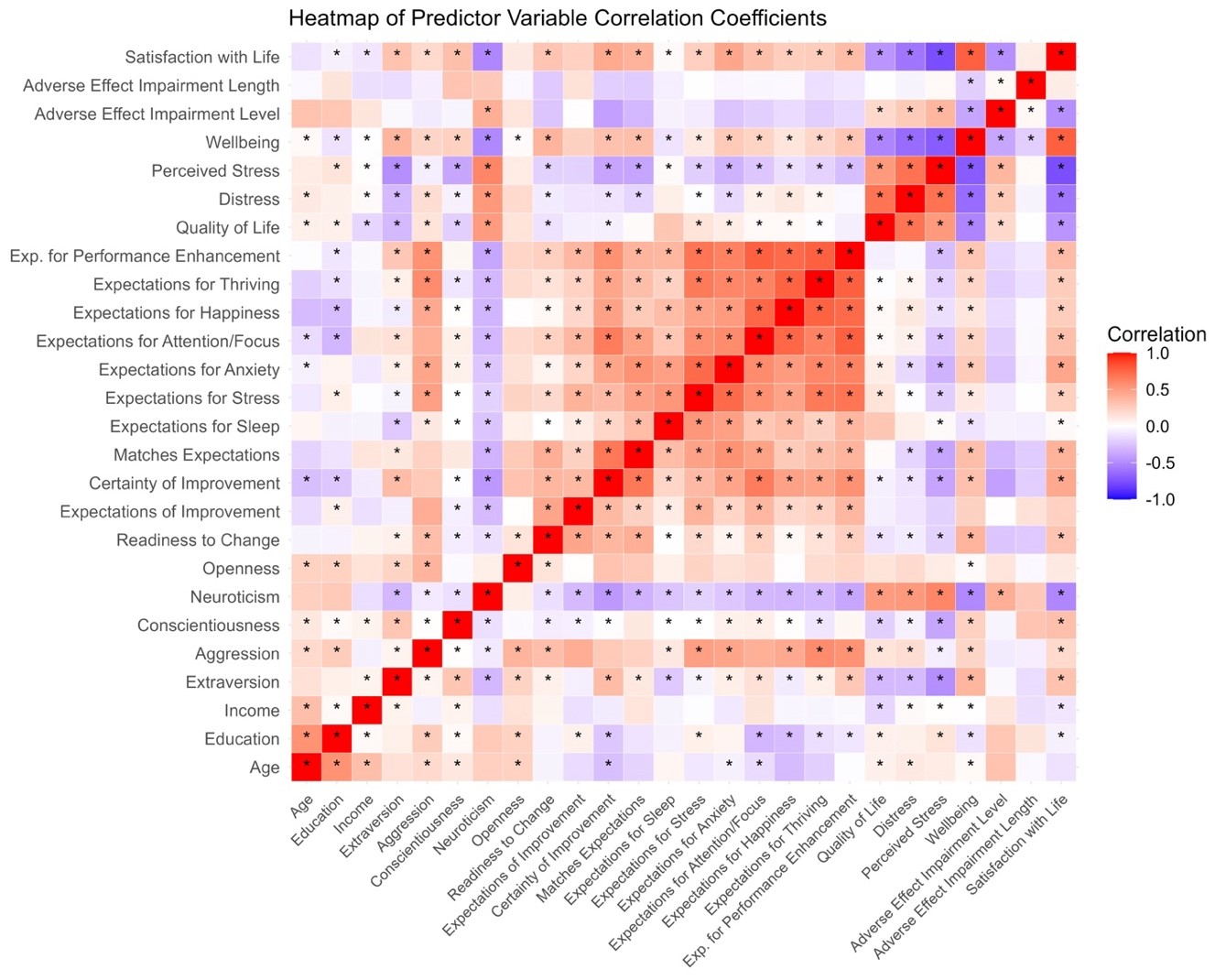


Figure S2.

*Correlation heat map showing correlation coefficients between predictor variables.*

**Note**: * indicates a p-value less than 0.05.

Supplementary 11. Principal Component Analysis (PCA)

**Summary of PCA Results: Mental Health Factors**

Table S10.

*Summary of Mental Health Factor PCA Results: Standard Deviation, Proportion of Variance, and Cumulative Proportion*

| **Component** | **Eigenvalue** | **% of Variance** | **Cumulative % of Variance** |
| --- | --- | --- | --- |
| 1 | 3.863 | 77.270 | 77.269 |
| 2 | 0.481 | 9.617 | 86.887 |
| 3 | 0.267 | 5.342 | 92.228 |
| 4 | 0.216 | 4.328 | 96.557 |
| 5 | 0.172 | 3.443 | 100 |

*Note.* PC = Principal Component. PCA = Principal Component Analysis. Proportion of Variance refers to the variance explained by each component, while Cumulative Proportion represents the cumulative variance explained up to that component

Table S11.

*PCA Factor Loadings for the Mental Health Principal Component.*

| Factor | PC1 | PC2 | PC3 | PC4 | PC5 |
| --- | --- | --- | --- | --- | --- |
| Satisfaction with Life | -0.813 | 0.511 | 0.269 | -0.067 | 0.040 |
| Wellbeing | -0.900 | 0.124 | -0.217 | 0.358 | 0.018 |
| Perceived Stress | 0.899 | -0.053 | 0.316 | 0.288 | 0.081 |
| Distress | 0.886 | 0.342 | -0.084 | 0.052 | -0.300 |
| Quality of Life | 0.886 | 0.307 | -0.211 | -0.042 | 0.273 |

*Note.* PC = Principal Component.

Table S12.

*Summary of Expectations PCA Results: Standard Deviation, Proportion of Variance, and Cumulative Proportion*

| **Component** | **Eigenvalue** | **% of Variance** | **Cumulative % of Variance** |
| --- | --- | --- | --- |
| 1 | 2.584 | 64.903 | 64.603 |
| 2 | 0.698 | 17.446 | 82.049 |
| 3 | 0.491 | 12.270 | 94.320 |
| 4 | 0.227 | 5.681 | 100 |

*Note:* PCA = Principal Component Analysis.

Table S13.

*PCA Factor Loadings for the Expectations Principal Component.*

| **Factor** | **PC1** | **PC2** | **PC3** | **PC4** |
| --- | --- | --- | --- | --- |
| Expectations for Sleep | -0.664 | 0.725 | 0.171 | 0.061 |
| Expectations for Stress | -0.903 | -0.059 | -0.216 | -0.366 |
| Expectations for Anxiety | -0.863 | -0.166 | -0.371 | 0.300 |
| Expectations for Attention/Focus | -0.760 | -0.375 | 0.529 | 0.042 |

*Note:* PCA = Principal Component Analysis.

Supplementary 12. Scatterplots comparing factor score with engagement level in total minutes (log) for all factors significant in the regression


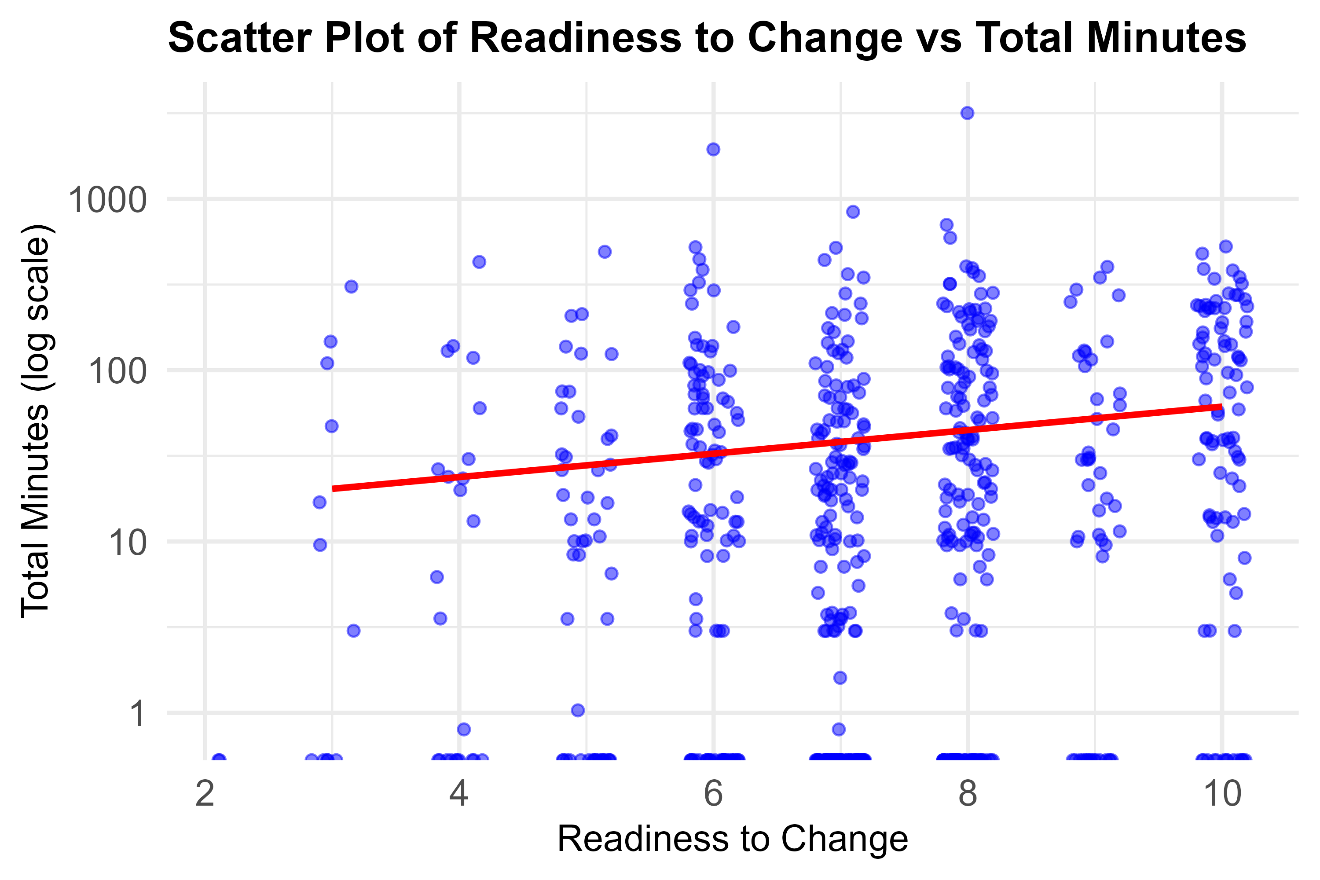


Figure S3.

*Scatter plot of Readiness to Change scores with total minutes.*


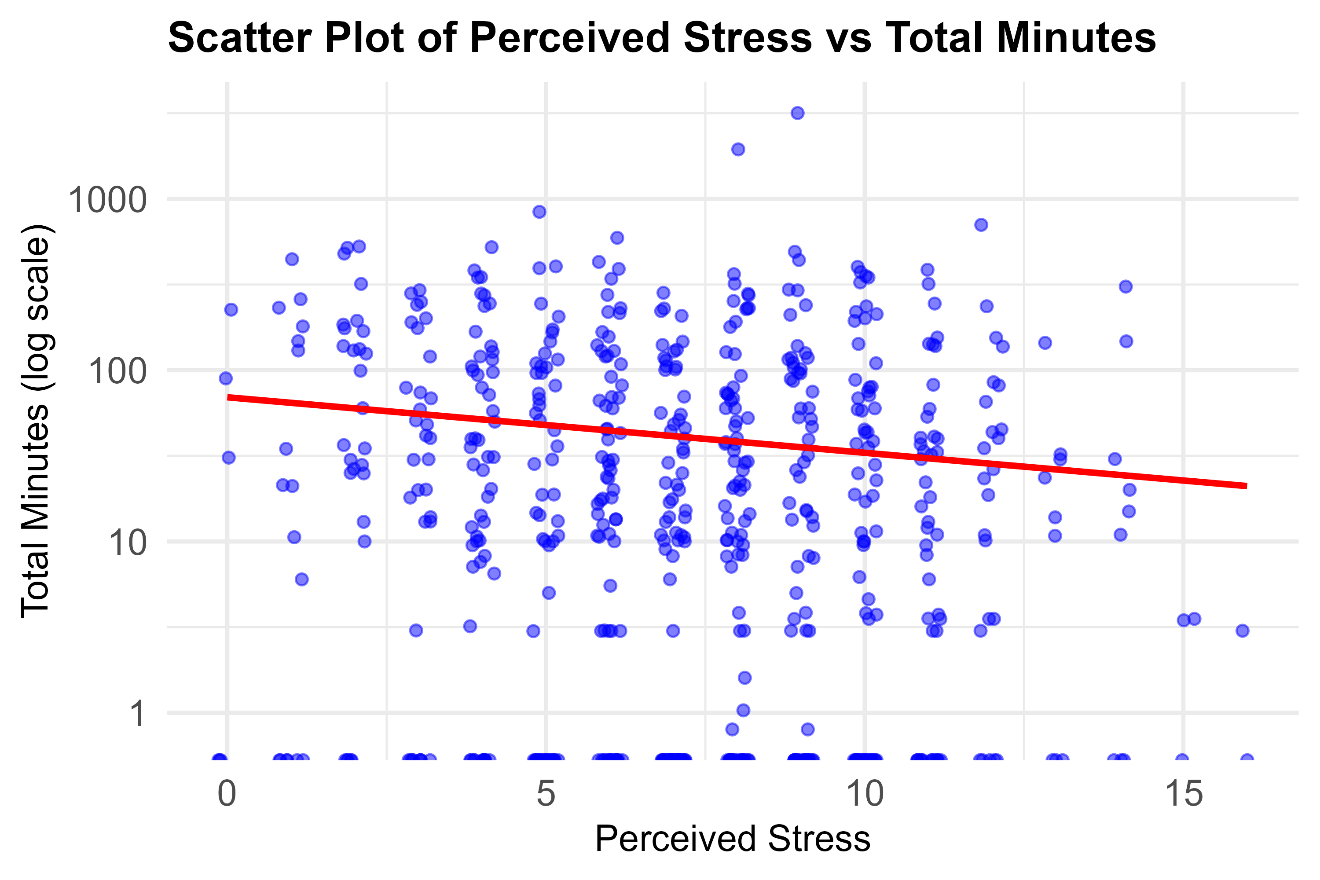


Figure S4.

*Scatter plot of Perceived Stress (PSS4) scores with total minutes.*


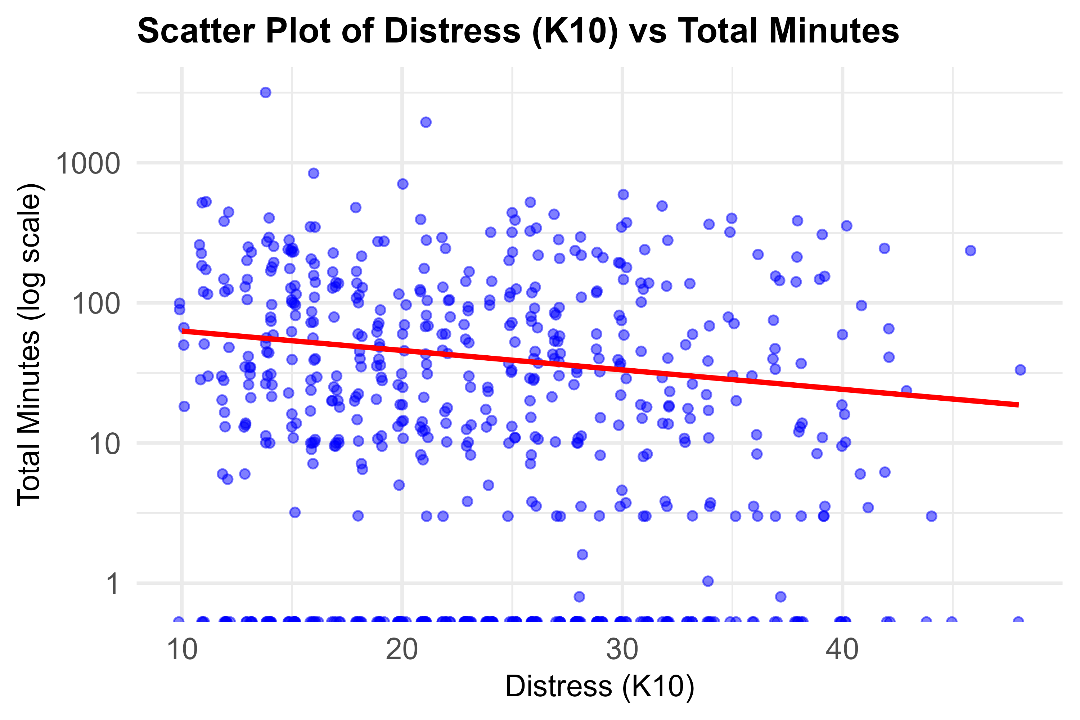


Figure S5.

*Scatter plot of Distress (K10) scores with total minutes.*


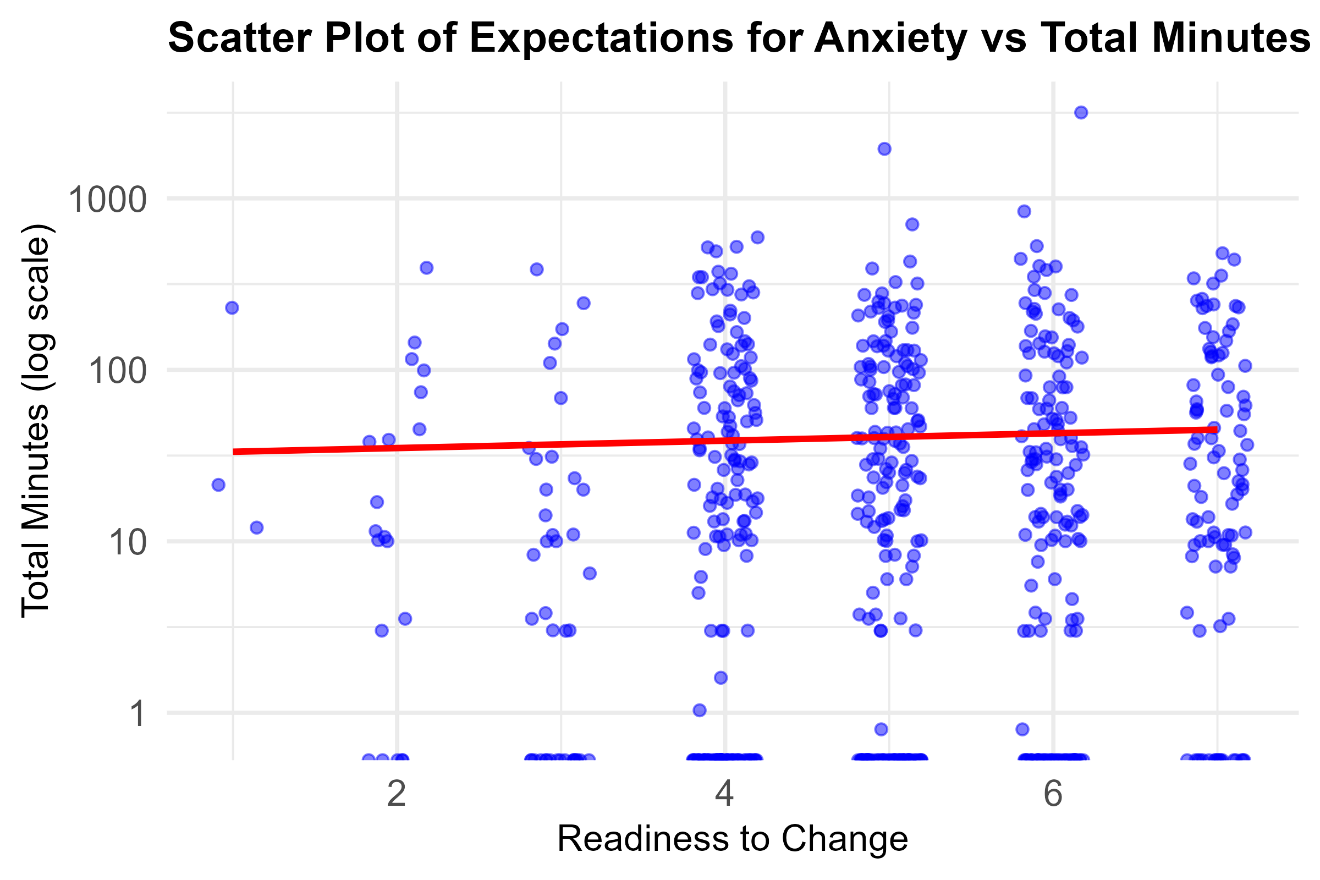


Figure S6.

*Scatter plot of Expectations for Anxiety scores with total minutes.*


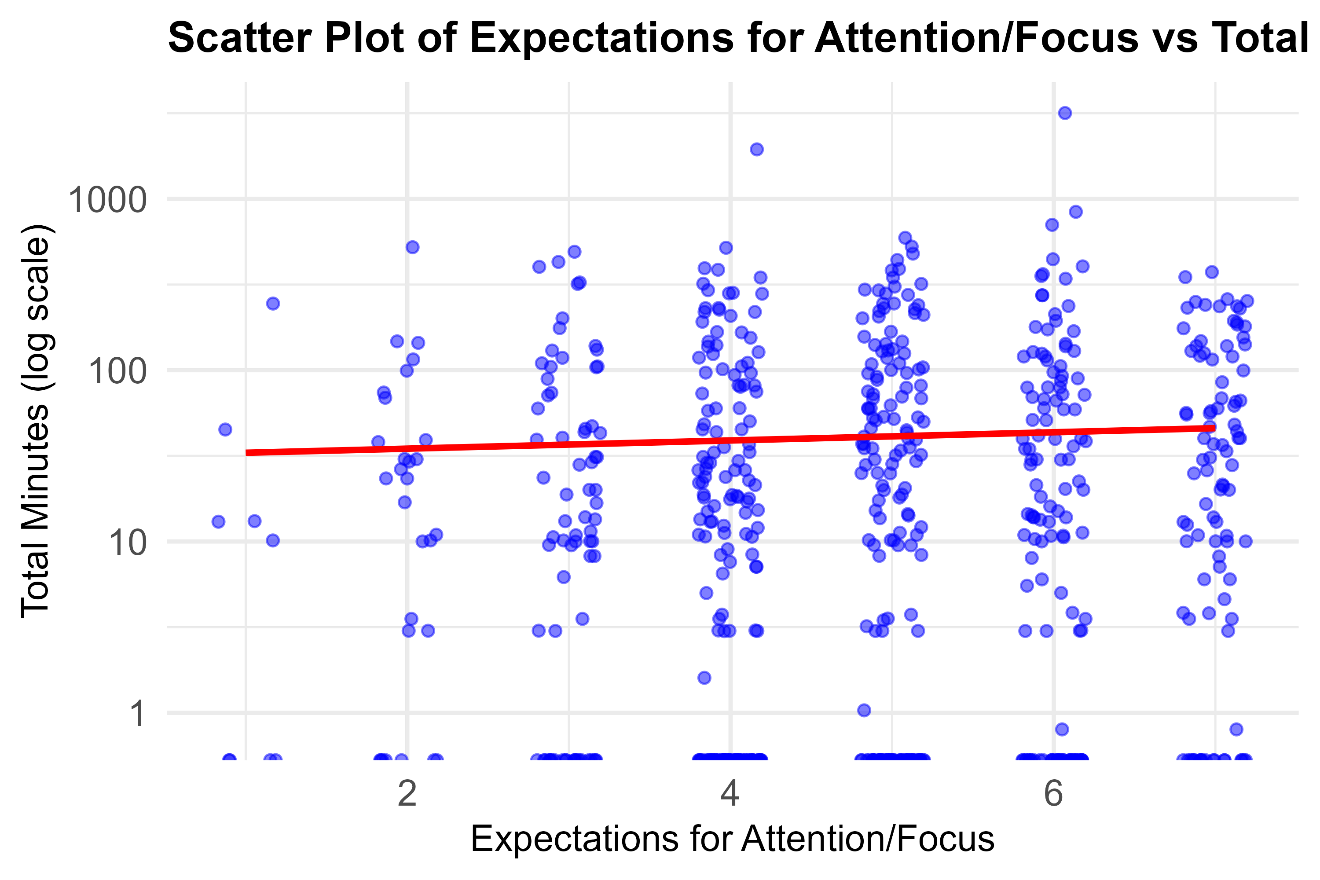


Figure S7.

*Scatter plot of Expectations for Attention/Focus scores with total minutes.*
